# Association between DNA mismatch repair gene polymorphisms and platinum-based chemotherapy toxicity in non-small cell lung cancer patients

**DOI:** 10.1186/s40880-016-0175-2

**Published:** 2017-01-16

**Authors:** Jun-Yan Liu, Chen-Yue Qian, Yuan-Feng Gao, Juan Chen, Hong-Hao Zhou, Ji-Ye Yin

**Affiliations:** 1 Xiangya School of Medicine, Central South University, Changsha, 410008 Hunan P. R. China; 2 Department of Clinical Pharmacology, Xiangya Hospital, Central South University, Changsha, 410008 Hunan P. R. China; 3 Institute of Clinical Pharmacology, Hunan Key Laboratory of Pharmacogenetics, Central South University, Changsha, 410078 Hunan P. R. China; 4 Hunan Province Cooperation Innovation Center for Molecular Target New Drug Study, Hengyang, 421001 Hunan P. R. China

**Keywords:** DNA mismatch repair, Lung cancer, Chemotherapy toxicity, Platinum

## Abstract

**Background:**

Chemotherapy toxicity is a serious problem from which non-small cell lung cancer (NSCLC) patients suffer. The mismatch repair (MMR) system is associated with platinum-based chemotherapy toxicity in NSCLC patients. In this study, we aimed to investigate the relationship between genetic polymorphisms in the MMR pathway and platinum-based chemotherapy toxicity in NSCLC patients.

**Methods:**

A total of 220 Chinese lung cancer patients who received at least two cycles of platinum-based chemotherapy were recruited for this study. Toxicity was evaluated in each patient after two cycles of chemotherapy. A total of 44 single nucleotide polymorphisms were selected to investigate their associations with platinum-based chemotherapy toxicity.

**Results:**

MutS homolog 2 (*MSH2*) rs6544991 [odds ratio (OR) 2.98, 95% confidence interval (CI) 1.20–7.40, *P* = 0.019] was associated with gastrointestinal toxicity in the dominant model; *MSH3* rs6151627 (OR 2.38, 95% CI 1.23–4.60, *P* = 0.010), rs6151670 (OR 2.05, 95% CI 1.07–3.93, *P* = 0.031), and rs7709909 (OR 2.38, 95% CI 1.23–4.64, *P* = 0.010) were associated with hematologic toxicity in the dominant model. Additionally, *MSH5* rs805304 was significantly associated with overall toxicity (OR 2.21, 95% CI 1.19–4.09, *P* = 0.012), and *MSH5* rs707939 was significantly associated with both overall toxicity (OR 0.42, 95% CI 0.23–0.76, *P* = 0.004) and gastrointestinal toxicity (OR 0.44, 95% CI 0.20–0.96, *P* = 0.038) in the dominant model.

**Conclusion:**

Genetic polymorphisms in the MMR pathway are potential clinical markers for predicting chemotherapy toxicity in NSCLC patients.

**Electronic supplementary material:**

The online version of this article (doi:10.1186/s40880-016-0175-2) contains supplementary material, which is available to authorized users.

## Background

Non-small cell lung cancer (NSCLC) is a common cancer and the main cause of cancer-related mortality worldwide [1–5]. Although many new target drugs, such as gefitinib and erlotinib, have been used to treat NSCLC, cytotoxic drugs, such as platinum, are still used as first-line agents in the treatment of NSCLC [6, 7]. However, adverse drug reactions, such as nephrotoxicity, hepatotoxicity, hematologic toxicity, and gastrointestinal toxicity, are major obstacles to successful treatment [8–10]. Thus, it is important to identify biomarkers that can be used to predict platinum-based chemotherapeutic toxicity [11].

DNA mismatch repair (MMR) is a key DNA repair system [12, 13]. It is highly conserved and plays an important role in correcting errors generated during DNA replication [14, 15]. MMR proteins interact with one another to form protein complexes that recognize and digest mismatched DNA segments and ultimately fill mismatch gaps [16]. Briefly, MutL homolog 1 (*MLH1*) dimerizes with postmeiotic segregation increased 1 (*PMS1*), *PMS2*, or *MLH3* to form the *MLH1*/*PMS2* (*MutLα*), *MLH1*/*PMS1* (*MutLβ*), or *MLH1*/*MLH3* (*MutLγ*) heterodimer and MutS homolog 2 (*MSH2*) dimerizes with *MSH6* or *MSH3* to form the *MSH2*/*MSH6* (*MutSα*) or *MSH2*/*MSH3* (*MutSβ*) heterodimer so as to bind the DNA helix and recognize DNA mismatches. Together with the abovementioned *MutL* complexes, the *MutSα* complex guides the repair of single-base and small-loop mismatches, whereas the *MutSβ* complex guides the repair of small- to large-loop mismatches [16–19]. Thus, *MutSα* and *MutLα* are key proteins in the MMR system and are responsible for mismatch detection and subsequent repair event coordination [20, 21].

There are several partially overlapping DNA repair pathways, including base excision repair (BER), nucleotide excision repair (NER), double-strand break repair (DSBR), and MMR [22]. A previous study showed that mutations in DNA repair genes affect the effectiveness and toxicity of platinum-based chemotherapy in NSCLC patients [23]. MMR plays a key role in maintaining genomic stability through the highly conserved biological pathway. It is an important determinant of platinum cellular toxicity. The formation of platinum/DNA adducts blocks replication and transcription of DNA, and the MMR system plays an important role in removing these adducts. MMR defects lead to replication and recombination errors and cause 6-thioguanine (6-TG)- and O6-methylguanine-induced toxicity in DNA glycosylase-deficient cells [24]. It has been reported that mutations in MMR pathway genes may be associated with platinum-based chemotherapy toxicity in NSCLC patients [23, 25]. In addition, mutations in MMR genes, particularly those in *MSH3* and *MSH5*, may be associated with the risk of lung cancer and even lead to increased alkylation tolerance [26].

Our previous studies showed that genetic polymorphisms were useful clinical markers for chemotherapy response and toxicity prediction in lung cancer patients [9, 27–29]. To investigate the relationship between MMR pathway genetic polymorphisms and platinum-induced toxicity, we evaluated 6 MMR genes (*MLH1*, *MSH2*, *MSH3*, *MSH4*, *MSH5*, and *MSH6*) in Chinese NSCLC patients.

## Methods

### Study subjects

All patients met the following inclusion criteria were selected: (1) patients between 18 and 80 years old; (2) patients newly diagnosed with NSCLC, including adenocarcinoma or squamous cell carcinoma, with histological or cytological examination at the Affiliated Cancer Hospital or Xiangya Hospital of Central South University (Changsha, Hunan, China) between December 2012 and December 2015; (3) patients who received at least two cycles of platinum-based chemotherapy, e.g., cisplatin or carboplatin chemotherapy; (4) patients with no history of chemotherapy or radiotherapy; and (5) patients with no history of surgery before or during chemotherapy. Patients with active infections or other concomitant malignancies were excluded.

All patients provided written informed consent before they participated in this study. The study protocol was approved by the Ethics Committee of Xiangya School of Medicine, Central South University (approval number: CTXY-110008-1). This clinical research project was approved by the Chinese Clinical Trial Registry under the following registration number: ChiCTR-RNC-12002892 (http://www.chictr.org/cn/).

### SNP selection, DNA extraction, and genotyping

All single nucleotide polymorphisms (SNPs) were selected by Haploview (Broad Institute, Cambridge, MA, USA) using pair-wise tagging with default settings (pair-wise *r* ^*2*^ threshold = 0.8). The following SNPs were eligible for further study: SNPs with a minor allele frequency (MAF) ≥ 5% in the Han Chinese population and SNPs in Hardy–Weinberg equilibrium (HWE) (*P* > 0.05).

All blood samples were collected in the morning and stored at −20°C for 4 h. Genomic DNA was isolated using a Genomic DNA Purification Kit (Promega, Madison, WI, USA) and stored at −20°C before use. Genotyping was conducted using a Sequenom MassARRAY Genotyping Platform (Sequenom, San Diego, CA, USA).

### Toxicity evaluation criteria

Platinum-based chemotherapy-induced toxicity was estimated according to the National Cancer Institute Common Toxicity Criteria, Version 3.0. The toxicity intensity was graded on a scale of 1–5 as follows: grade 1, mild adverse events; grade 2, moderate adverse events; grade 3, severe adverse events; grade 4, life-threatening or disabling adverse events; and grade 5, death related to adverse events. We recruited patients experiencing grade 0 to grade 4 toxicity, and they were divided into two categories. Patients experiencing grade 0–2 adverse events were classified into the low-toxicity category, whereas patients with grades 3 and 4 adverse events were classified into the severe toxicity category.

### Statistical analysis

The genotype frequencies observed among all patients were compared with their expected frequencies under Hardy–Weinberg equilibrium using a χ^2^ test (*P* > 0.05). Sex, age, smoking status, tumor histology, clinical stage, and Eastern Cooperative Oncology Group (ECOG) performance status were considered potential covariates for logistic regression. All analyses were performed using PLINK (version 1.07, http://pngu.mgh.harvard.edu/purcell/plink/) and SPSS 13.0 software (SPSS Inc, Chicago, IL, USA). Odds ratios (OR) and their 95% confidence intervals (95% CI) were used to assess the association between treatment outcomes and gene polymorphisms. *P* < 0.05 was considered statistically significant.

## Results

### Patient characteristics

A total of 220 patients who received first-line platinum-based chemotherapy were recruited for this study. A total of 44 SNPs were genotyped in these patients, and 37 of them were in HWE (*P* > 0.05) and exhibited an MAF ≥ 5%. The basic information of these SNPs and the clinical characteristics of these lung cancer patients are summarized in Tables 1 and 2, respectively. Hematologic, gastrointestinal, and overall toxicity were assessed after the first two cycles of chemotherapy. Severe overall toxicity occurred in 79 (35.9%) patients. Among them, severe hematologic and gastrointestinal toxicity occurred in 55 (25.0%) and 31 (14.1%) patients, respectively.

**Table 1:** Thirty-seven single nucleotide polymorphisms (SNPs) in DNA mismatch repair (MMR) genes

Gene	SNP (rs number)	Allele	Localization	Call rate (%)	MAF	HWE
*MLH1*	rs10849	G/A	3′-UTR	99.55	0.09	0.764
	rs1540354	A/T	Intron	97.73	0.68	0.635
	rs749072	T/C	Intron	97.73	0.62	0.217
*MSH2*	rs10191478	T/G	Intron	97.73	0.20	0.264
	rs12999145	A/G	Intron	98.64	0.53	0.783
	rs13019654	G/T	Intron	96.82	0.28	0.710
	rs1981929	G/A	Intron	97.27	0.86	0.309
	rs2303428	A/C	Intron	98.64	0.33	0.234
	rs4608577	T/G	Intron	93.18	0.13	0.106
	rs4952887	C/T	Intron	98.18	0.15	0.124
	rs6544991	A/C	Intron	95.00	0.36	0.605
	rs7602094	T/C	Intron	95.91	0.66	0.560
*MSH3*	rs245340	A/C	Intron	97.73	0.24	0.203
	rs245346	T/C	Intron	97.73	0.45	0.738
	rs26778	A/T	Intron	96.36	0.59	0.713
	rs26784	T/C	Intron	97.73	0.37	0.860
	rs3816729	A/G	Intron	96.36	0.30	0.927
	rs6151627	A/G	Intron	99.09	0.29	0.554
	rs6151670	C/G	Intron	98.18	0.28	0.258
	rs6151892	T/A	Intron	99.09	0.33	0.729
	rs6151914	C/T	Intron	97.73	0.09	0.065
	rs7709909	C/T	Intron	99.09	0.31	0.572
*MSH4*	rs3806162	T/G	5′ near gene	99.09	0.22	0.393
	rs5745532	T/C	Intron	99.09	0.75	0.112
*MSH5*	rs3117572	G/A	Intron	98.64	0.28	0.103
	rs409558	A/G	Intron	100.00	0.13	0.312
	rs707937	C/G	Intron	96.82	0.42	0.369
	rs707938	A/G	Synonymous	97.27	0.30	0.428
	rs707939	G/T	Intron	100.00	0.39	0.097
	rs805304	C/A	5′ near gene	98.18	0.69	0.448
*MSH6*	rs2020910	T/A	Intron	99.09	0.17	0.893
	rs2348244	T/C	Intron	99.09	0.39	0.856
	rs2710163	T/C	Intron	98.18	0.70	0.856
	rs3136329	T/C	Intron	97.27	0.13	0.108
	rs3732190	G/A	Intron	95.91	0.09	0.933
	rs6713506	G/A	Intron	98.64	0.06	0.852
	rs6742522	G/A	Intron	99.09	0.11	0.259

*MLH1* MutL homolog 1, *MSH2*-*6* MutS homolog 2-6, *A* adenine, *T* thymine, *C* cytosine, *G* guanine, *UTR* untranslated region, *MAF* minor allele frequency, *HWE* Hardy–Weinberg equilibrium

**Table 2:** The clinical characteristics of the 220 non-small cell lung cancer (NSCLC) patients

Variate	Number of patients [cases (%)]
Total	220
Age (years)	
≤55	93 (42.3)
>55	127 (57.7)
Smoking status	
Never	95 (43.2)
Ever	125 (56.8)
Gender	
Male	165 (75.0)
Female	55 (25.0)
ECOG PS	
0–1	39 (17.7)
2	181 (82.3)
Histological type	
Adenocarcinoma	108 (49.1)
Squamous cell carcinoma	112 (50.9)
Stage	
I–II	8 (3.6)
III–IV	212 (96.4)
Platinum-based drug	
Cisplatin	37(16.8)
Carboplatin	183 (83.2)
Chemotherapy regimen	
Platinum-gemcitabine	112 (50.9)
Platinum-pemetrexed	68 (30.9)
Platinum-paclitaxel	23 (10.4)
Platinum-docetaxel	12 (5.5)
Platinum-navelbine	5 (2.3)
Severe toxicity	
Total	79 (35.9)
Hematologic toxicity	55 (25.0)
Gastrointestinal toxicity	31 (14.1)

*ECOG* Eastern Cooperative Oncology Group, *PS* performance status

### Association between MMR gene polymorphisms and toxicity

The genotypes of the 37 SNPs in 6 DNA MMR genes were determined in the 220 patients. The results are summarized in Additional file 1: Table S1. Six SNPs exhibited significant associations with toxicity (Table 3). *MSH2* rs6544991 (OR 2.98, 95% CI 1.20–7.40, *P* = 0.019) was associated with gastrointestinal toxicity in the dominant model. *MSH3* rs6151627 (OR 2.38, 95% CI 1.23–4.60, *P* = 0.010), *MSH3* rs6151670 (OR 2.05, 95% CI 1.07–3.93, *P* = 0.031), and *MSH3* rs7709909 (OR 2.38, 95% CI 1.23–4.64, *P* = 0.010) were associated with hematologic toxicity in the dominant model. *MSH5* rs805304 was significantly associated with overall toxicity (additive model: OR 1.66, 95% CI 1.04–2.65, *P* = 0.033; dominant model: OR 2.21, 95% CI 1.19–4.09, *P* = 0.012). *MSH5* rs707939 was significantly associated with overall toxicity (additive model: OR 0.45, 95% CI 0.28–0.73, *P* = 0.001; dominant model: OR 0.42, 95% CI 0.23–0.76, *P* = 0.004; recessive model: OR 0.27, 95% CI 0.09–0.84, *P* = 0.023) and gastrointestinal toxicity (additive model: OR 0.46, 95% CI 0.24–0.88, *P* = 0.020; dominant model: OR 0.44, 95% CI 0.20–0.96, *P* = 0.038).

**Table 3:** Associations between MMR gene polymorphisms and platinum-based chemotherapy toxicity in the 220 NSCLC patients

Toxicity	Gene	SNP	Additive model		Dominant model		Recessive model	
			OR (95% CI)	*P*	OR (95% CI)	*P*	OR (95% CI)	*P*
Overall	*MSH2*	rs6544991	1.18 (0.77–1.79)	0.447	1.39 (0.76–2.55)	0.290	1.02 (0.44–2.33)	0.973
	*MSH3*	rs6151627	1.20 (0.77–1.86)	0.419	1.49 (0.83–2.65)	0.181	0.77 (0.27–2.17)	0.618
		rs6151670	1.14 (0.73–1.76)	0.572	1.37 (0.76–2.45)	0.293	0.75 (0.27–2.13)	0.593
		rs7709909	1.21 (0.79–1.87)	0.380	1.51 (0.84–2.71)	0.163	0.83 (0.31–2.19)	0.703
	*MSH5*	rs707939	0.45 (0.28–0.73)	0.001	0.42 (0.23–0.76)	0.004	0.27 (0.09–0.84)	0.023
		rs805304	1.66 (1.04–2.65)	0.034	2.21 (1.19–4.09)	0.012	1.21 (0.44–3.34)	0.716
Hematologic	*MSH2*	rs6544991	1.01 (0.64–1.61)	0.957	0.97 (0.50–1.87)	0.919	1.12 (0.45–2.77)	0.805
	*MSH3*	rs6151627	1.55 (0.96–2.50)	0.074	2.38 (1.23–4.60)	0.010	0.75 (0.23–2.44)	0.635
		rs6151670	1.42 (0.88–2.29)	0.153	2.05 (1.07–3.93)	0.032	0.75 (0.23–2.43)	0.630
		rs7709909	1.55 (0.97–2.49)	0.067	2.38 (1.23–4.64)	0.010	0.87 (0.30–2.58)	0.808
	*MSH5*	rs707939	0.68 (0.41–1.12)	0.128	0.66 (0.35–1.28)	0.219	0.50 (0.16–1.54)	0.225
		rs805304	1.37 (0.83–2.27)	0.223	1.99 (1.01–3.90)	0.047	0.60 (0.16–2.20)	0.436
Gastrointestinal	*MSH2*	rs6544991	1.56 (0.90–2.69)	0.113	2.98 (1.20–7.40)	0.019	0.88 (0.28–2.76)	0.827
	*MSH3*	rs6151627	0.63 (0.33–1.20)	0.161	0.54 (0.24–1.19)	0.128	0.66 (0.14–3.02)	0.592
		rs6151670	0.67 (0.35–1.27)	0.220	0.59 (0.27–1.32)	0.199	0.64 (0.14–2.94)	0.568
		rs7709909	0.62 (0.33–1.18)	0.145	0.55 (0.25–1.20)	0.132	0.58 (0.13–2.63)	0.481
	*MSH5*	rs707939	0.46 (0.24–0.88)	0.020	0.44 (0.20–0.96)	0.038	0.21 (0.03–1.65)	0.139
		rs805304	1.64 (0.90–2.97)	0.105	1.86 (0.83–4.20)	0.133	1.85 (0.56–6.14)	0.314

*OR* odds ratio, *95% CI* 95% confidence interval; other abbreviations as in Table 1

### Stratification analyses

**Fig. 1:**
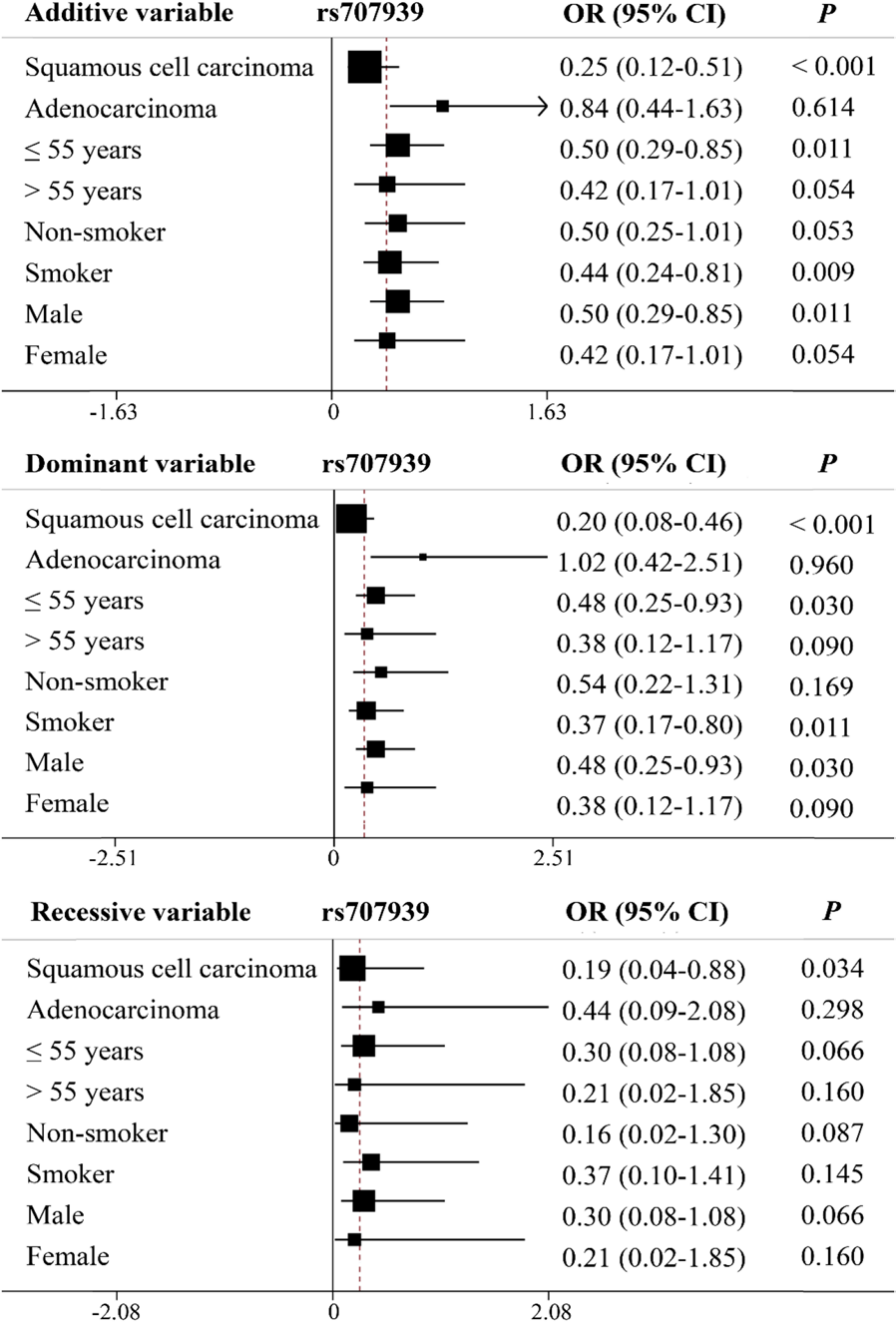
Stratification analyses of the associations between the MutS homolog 5 (*MSH5*) rs707939 polymorphism and overall platinum-based chemotherapy toxicity in the 220 non-small cell lung cancer patients using the additive, dominant, and recessive models. *Each box* and *horizontal line* represents an odds ratio (OR) and a 95% confidence interval (CI)

Stratification analyses were performed to investigate the associations between all SNPs that were significantly associated with overall toxicity. Patients were stratified by cancer type (squamous cell carcinoma or adenocarcinoma), age (≤55 years or >55 years), smoking status (non-smoker or smoker), and sex (male or female). As shown in Fig. 1, *MSH5* rs707939 exhibited significant associations with squamous cell carcinoma (additive model: OR 0.25, 95% CI 0.12–0.51, *P* < 0.001; dominant model: OR 0.20, 95% CI 0.08–0.46, *P* < 0.001; recessive model: OR 0.19, 95% CI 0.04–0.88, *P* = 0.034), patients ≤55 years of age (additive model: OR 0.50, 95% CI 0.29–0.85, *P* = 0.011; dominant model: OR 0.48, 95% CI 0.25–0.93, *P* = 0.030), smokers (additive model: OR 0.44, 95% CI 0.24–0.81, *P* = 0.009; dominant model: OR 0.37, 95% CI 0.17–0.80, *P* = 0.011), and male patients (additive model: OR 0.50, 95% CI 0.29–0.85, *P* = 0.011; dominant model: OR 0.48, 95% CI 0.25–0.93, *P* = 0.030). No significant associations were noted for any other SNPs. Taken together, these results indicate that T allele carriers of *MSH5* rs707939 polymorphism have better tolerance to gastrointestinal toxicity and overall toxicity than carriers of other polymorphisms.

## Discussion

In this study, we investigated whether polymorphisms of MMR genes (*MLH1*, *MSH2*, *MSH3*, *MSH4*, *MSH5*, and *MSH6*) were associated with platinum-based chemotherapy toxicity in 220 NSCLC patients. We evaluated the associations between these gene polymorphisms and gastrointestinal, hematologic, and overall toxicities. Our results showed that *MSH2* rs6544991 was associated with gastrointestinal toxicity, *MSH3* rs6151627, rs6151670, and rs7709909 were associated with hematologic toxicity, and *MSH5* rs707939 and rs805304 were associated with gastrointestinal toxicity and overall toxicity.

A previous study showed that MSH2 was a key protein that influenced 6-thioguanine (6-TG)- and O^6^-methylguanine-induced toxicity in DNA glycosylase-deficient cells, indicating that *MSH2* plays an important role in attenuating oxidative DNA damage [24]. In our study, C allele carriers of rs6544991, which features an A/C single-nucleotide variation located in the intron area of *MSH2*, exhibited poor gastrointestinal toxicity tolerance after being treated with platinum-based chemotherapy. We speculated that this SNP may affect the ability of *MSH2* to remove platinum adducts. In addition, our results showed that *MSH3* rs6151627 G allele carriers, rs6151670 G allele carriers, and rs7709909 T allele carriers exhibited poor hematologic toxicity tolerance after being treated with platinum-based chemotherapy. *MSH3* rs6151627 is an A/G single-nucleotide variation, rs6151670 is a C/G single-nucleotide variation, and rs7709909 is a C/T single-nucleotide variation. All of these polymorphisms are intron variants of *MSH3*. Methylation of the *MSH3* promoter is involved in esophageal tumorigenesis, suggesting that it plays an important role in modulating cell chemosensitivity. As a DNA MMR gene, *MSH3* forms the *MutSβ* heteroduplex with *MSH2*. The *MSH2*/*MSH3* heterodimer is an ATPase that plays a critical role in mismatch recognition and repair initiation. It binds to DNA mismatches by recognizing 2- to 13-bp insertion-deletion loops [30]. The three SNPs are speculated to affect the function of *MSH3*; however, the underlying mechanism is still unclear.

Another important finding of our study was that *MSH5* polymorphisms were significantly related to overall toxicity. *MSH5* rs707939 T allele carriers exhibited better gastrointestinal and overall toxicity tolerance after being treated with platinum-based chemotherapy. Moreover, among *MSH5* rs707939 T allele carriers, stratification analysis showed that male patients, patients ≤55 years old, smokers, and patients diagnosed with squamous cell carcinoma faced a lower risk of overall severe toxicity than their counterparts. All these results indicated that *MSH5* rs707939 was associated with reduced cisplatin-induced gastrointestinal and overall toxicities in NSCLC patients. In addition, *MSH5* rs805304 was also significantly associated with gastrointestinal and overall toxicities. It is noteworthy that rs707939 is a G/T single-nucleotide variation in the intron of *MSH5*. Previous studies suggested that mutations in *MSH5* result in alkylation tolerance in mammalian cells, which is associated with lung cancer risk [26, 31]. Thus, rs707939 and rs805304 may also affect MSH5 activity.

As far as we know, DNA MMR gene defects lead to MMR function loss, which increases the spontaneous mutation frequencies of cells. Cell mutation phenotypes are thought to result in malignant transformation and cause continuous accumulation of gene mutation events. Many error messages across the entire genome eventually affect the effectiveness and toxicity of chemotherapy. However, the detailed mechanisms underlying the effects of these SNPs on gene function need to be studied further.

## Conclusions

Our findings indicate that carriers of the *MSH5* rs707939 T allele, the *MSH2* rs6544991 C allele, the *MSH3* rs6151627 and rs6151670 G alleles, and the *MSH3* rs7709909 T allele have poor toxicity tolerance. Therefore, these polymorphisms are potential clinical markers for predicting platinum-based chemotherapy toxicity in Chinese NSCLC patients. However, a study with a larger sample size is needed to validate these findings in the future.

## Additional file

**Additional file 1: Table S1.** Associations between 37 MMR SNP and platinum-based chemotherapy toxicity (Original Dates Analysis).
